# Shop till you drop

**DOI:** 10.1007/s12471-020-01479-x

**Published:** 2020-07-31

**Authors:** L. Baris, E. J. van den Bos

**Affiliations:** Department of Cardiology, Albert Schweitzer Hospital, Dordrecht, The Netherlands

## Answer

**Fig. 1:**
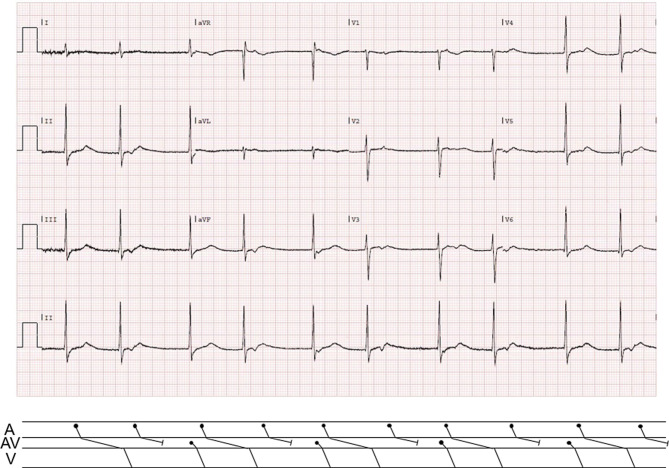
Electrocardiogram at presentation

The phenomenon of grouped beating is present, in which the QRS complexes occur in groups of two beats and the cycle length between the groups is less than twice the RR interval within the groups. The second beat of each group is preceded by a P wave, with a very long PR interval of 680 msec, whereas the first beat comprises a junctional escape (Fig. 1). This could be mistaken for a sinus bradycardia with first-degree atrioventricular (AV) block and premature junctional depolarisations in bigeminy. However, the extreme PR interval makes it more plausible that one of these P waves is blocked at the level of the AV node.

Thus, the diagnosis is a second-degree 2:1 AV block with an extremely long PR interval in a healthy young man due to increased vagal tone. The very long PR interval could be exaggerated due to increased refractoriness of the AV node caused by retrograde conduction of the junctional escape beat or due to slow pathway conduction in dual AV nodal physiology, or a combination of the two.

Vagally mediated AV block frequently occurs because of a vagal surge and is generally a benign physiological phenomenon in healthy young individuals [1].

## References

[1] Alboni P, Holz A, Brignole M (2013). Vagally mediated atrioventricular block: pathophysiology and diagnosis. Heart.

